# Supplementary material for: Inferring B cell phylogenies from paired heavy and light chain BCR sequences with Dowser
Source: bioRxiv. 2023 Oct 2:2023.09.29.560187. Preprint. [Version 1] doi: 10.1101/2023.09.29.560187 (PMC10592837; doi:10.1101/2023.09.29.560187)
Supplement: 1 [file NIHPP2023.09.29.560187V1-supplement-1.pdf]

bioRxiv preprint doi: https://doi.org/10.1101/2023.09.29.560187; this version posted October 2, 2023. The copyright holder for this preprint (which was not certified by peer review) is the author/funder, who has granted bioRxiv a license to display the preprint in perpetuity. It is made available under aCC-BY 4.0 International license.

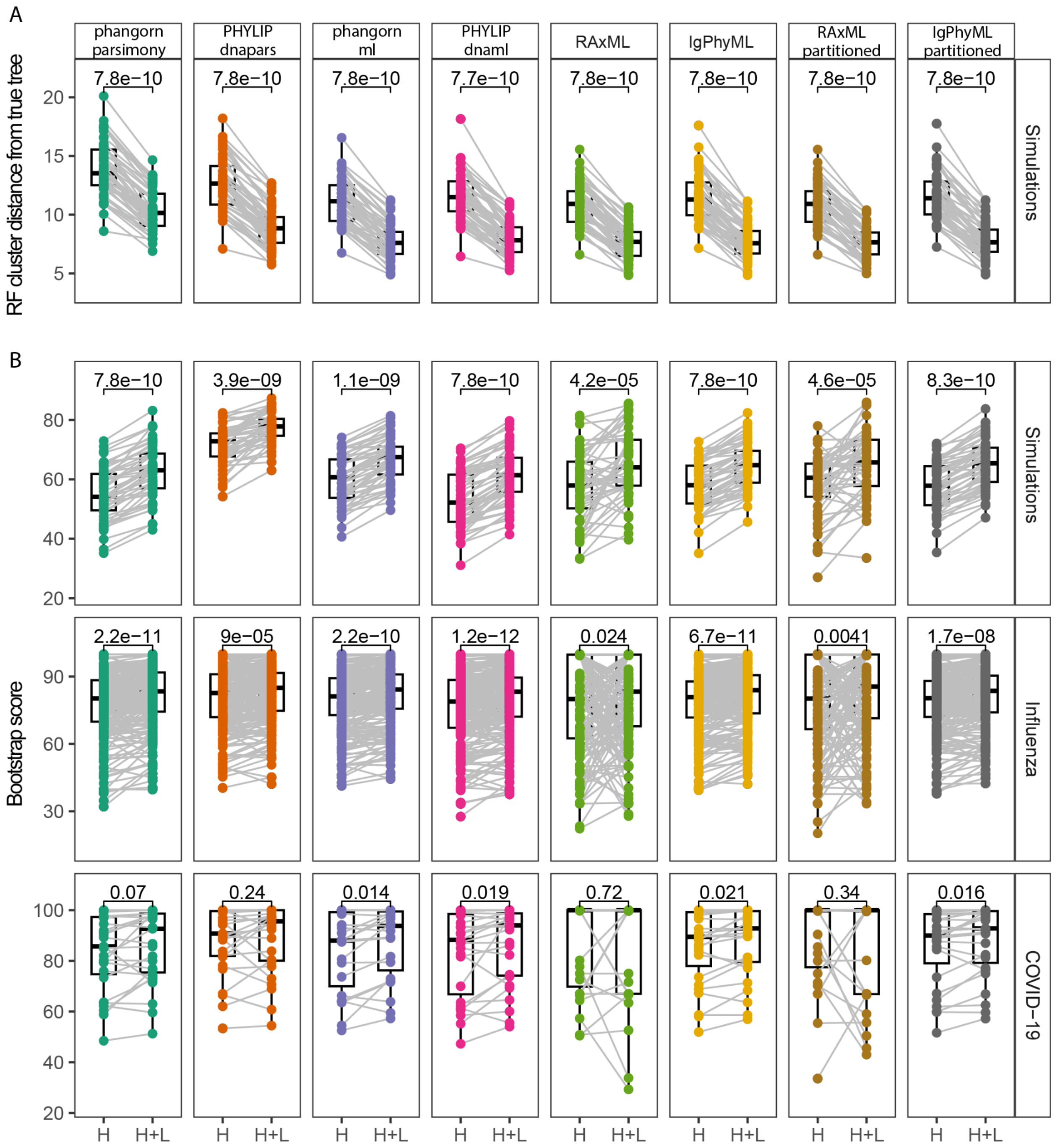

Supplementary Figure 1. The accuracy and reproducibility of tree reconstruction are improved using paired heavy and light chains. Similar to **Fig. 1**, but showing boxplots for all eight methods tested. A) Robinson-Foulds (RF) cluster distance between estimated and true tree topologies for trees built using only the heavy chain (H) and paired heavy and light chain sequences (H+L). B) Similar, but with the mean bootstrap value for trees in each dataset. P values were calculated using a Wilcoxon test.

bioRxiv preprint doi: https://doi.org/10.1101/2023.09.29.560187; this version posted October 2, 2023. The copyright holder for this preprint (which was not certified by peer review) is the author/funder, who has granted bioRxiv a license to display the preprint in perpetuity. It is made available under aCC-BY 4.0 International license.

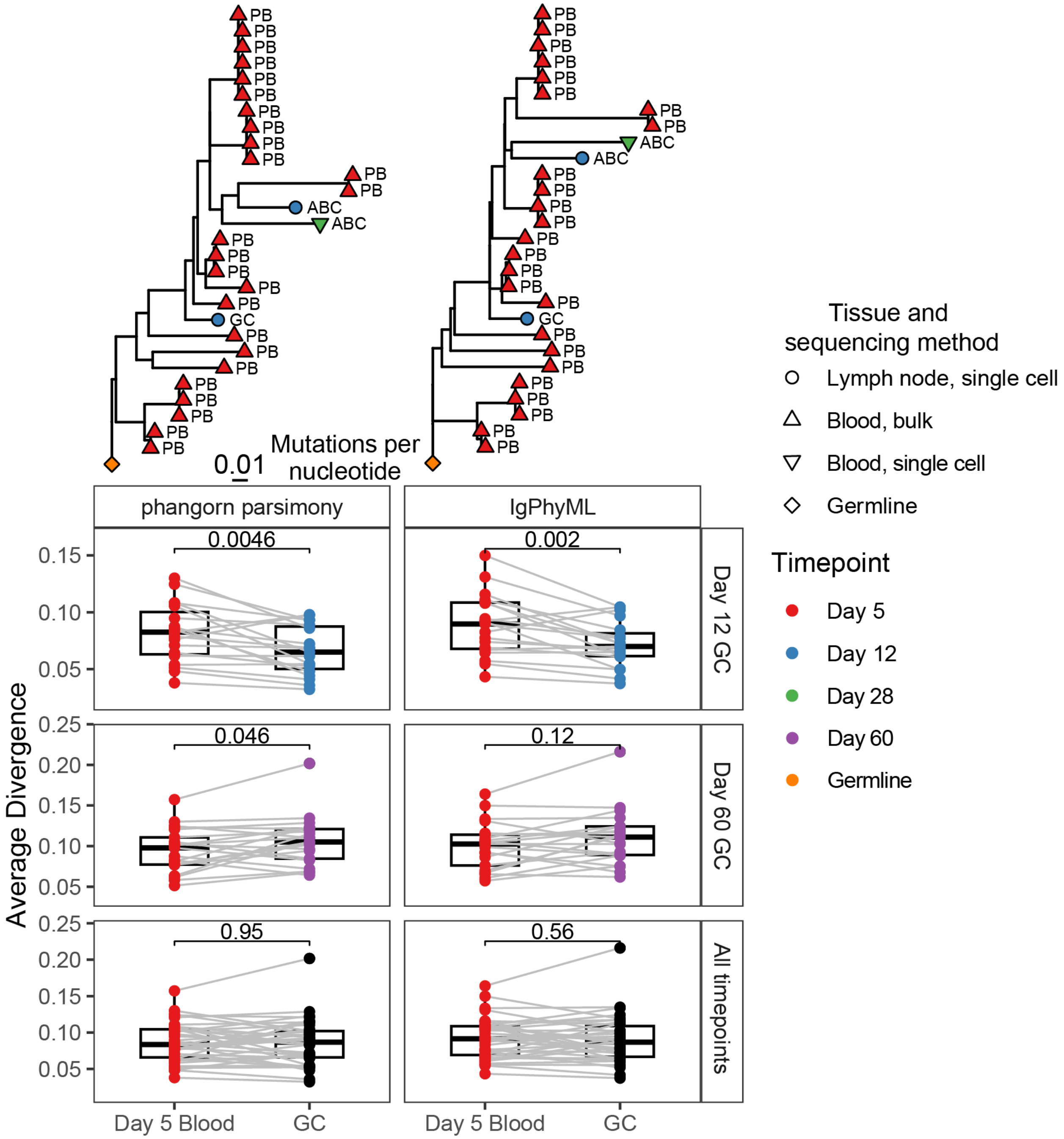

Supplementary Figure 2. Similar to **Fig. 5**, but using only heavy chain sequences. Multi-partition maximum likelihood methods were not tested because only heavy chain sequences were included in these comparisons. Above each plot is a representative tree.
